# Murine neutrophils present Class II restricted antigen

**DOI:** 10.1016/j.imlet.2008.02.008

**Published:** 2008-06-15

**Authors:** Shauna Culshaw, Owain R. Millington, James M. Brewer, Iain B. McInnes

**Affiliations:** Centre for Rheumatic Diseases, Glasgow Biomedical Research Centre, University of Glasgow, 120 University Place, Glasgow G12 8TA, UK

**Keywords:** Murine, Neutrophil, Antigen presentation

## Abstract

Neutrophils were originally described as short lived, terminally differentiated phagocytes that contribute only to the innate immune response. Recent evidence of neutrophil cytokine production and expression of numerous cell surface proteins has suggested that neutrophils are likely to influence adaptive responses and may satisfy the criteria of antigen presenting cells. Under certain inflammatory conditions human neutrophils express major histocompatibilty complex (MHC) Class II and the costimulatory molecules CD80 and CD86. We have employed a murine T cell hybridoma with a transgenic T cell receptor specific for ovalbumin peptide 323–339 (OVA_323–339_), and a green fluorescent reporter of T cell receptor ligation, to directly investigate neutrophil-T cell interactions. These cells provide an ideal model system, allowing precise identification of antigen specificity and a clear readout of T cell activation. Additionally, whilst murine neutrophils have previously been shown to stimulate MHC Class I-dependent CD8^+^ T cell activation, CD4^+^ T cells stimulation via MHC Class II-expressing neutrophils has not been investigated. We addressed this by isolating murine neutrophils, loading with OVA_323–339_ and co-culturing with T cells specific for the OVA_323–339_/MHC Class II complex, and this resulted in T cell activation, as determined by expression of the green-fluorescent protein reporter. Antigen-pulsed neutrophils were also able to prime naïve OVA-specific CD4^+^ T cells in a contact-dependent manner, as shown by proliferation and cytokine production. Activation of lymphocytes was not due to contaminating macrophages. These studies demonstrate that murine neutrophils present MHC Class II-restricted peptides and induce T cell proliferation, confirming findings in human neutrophils, and demonstrate a novel pro inflammatory effect of murine neutrophils.

## 1 Introduction

The range of functions ascribed to neutrophils has expanded significantly, from terminally differentiated, short-lived phagocytes that are solely involved in innate immune responses to cells that are now believed to influence the development of adaptive immune responses. Emerging evidence indicates that neutrophils may communicate with T cells through direct cell contact via major histocompatibility complex (MHC) Class I, Class II [1,2], and co-stimulatory molecules [3,4]. Additionally, a neutrophil-derived cytokine milieu [5] may have significant impact on the adaptive response to infection, for example via neutrophil-derived IL-12 [6,7].

There is considerable evidence to suggest that neutrophils satisfy the criteria of antigen presenting cells, with some capacity to process antigen. Human neutrophils express MHC Class II, either following in vitro activation via CD11b [8,9], with IFNγ, and GM-CSF [10,11] or following in vivo activation in RA synovial fluid [4,12], in patients with active Wegener's Granulomatosis [13], in patients receiving GM-CSF [14], and in persistent localized *Staphylococcus aureus* infections [15]. In addition to MHC Class II expression, stimulation of normal human PMN with IFNγ, or via CD11b induces CD80 and CD86 expression [8,11]. Human neutrophil presentation of superantigens is documented [2]. However, there are conflicting reports of neutrophils’ capacity to process and present tetanus toxoid on MHC Class II and activate T cells [1,2]. A capacity for extracellular antigen processing had been suggested as neutrophil gelatinase may cleave collagen to a Class II restricted immunodominant epitope [16].

Harding et al. demonstrated murine neutrophil Class I restricted antigen presentation [17] and additionally show that neutrophils processed phagocytosed bacteria via an alternate MHC Class I antigen-processing pathway, and such neutrophils may ‘regurgitate’ processed peptide into the extracellular space and this peptide may then bind MHC Class I on neighbouring cells (e.g. macrophages) for presentation to CD8 cells [17].

Human studies of antigen-specific neutrophil-T cell interactions are confounded by low frequency of antigen-specific T cells in peripheral blood, variation of MHC allele expression, which therefore impedes ideal peptide antigen identification. To overcome these complications, we have employed a murine model system. Moreover, despite the important role of the neutrophil in the pathogenesis of numerous inflammatory diseases, many of which are modelled in rodents, there is a relative paucity of information on murine Class II restricted antigen presentation. We, therefore, sought to investigate whether murine neutrophils present antigens via MHC Class II.

## 2 Materials and methods

### 2.1 Murine peritoneal neutrophil and macrophage separation

Peritoneal exudate cells (PEC) were obtained as previously described [18]. Briefly, 6–8-week-old BALB/c mice (Harlan Olac, Bicester, UK) received 1.5 ml i.p. Brewers thioglycollate medium (Difco, East Molesey, UK) and 4 h later animals were sacrificed and cells harvested by peritoneal lavage. PEC were washed once then resuspended in 8 ml PBS, and layered over 3 ml Histopaque^®^ 1083 (Sigma, Poole, UK), then centrifuged at 700 × *g* for 30 min at 20 °C. The enriched population of neutrophils contained within the cell pellet was washed twice in RPMI (Invitrogen, Paisley, UK) then resuspended at 2 × 10^6^ ml^−1^ in medium supplemented with 2 mM l-glutamine, 100 IU penicillin, 100 μg/ml streptomycin (all Invitrogen) and 10% foetal calf serum (Harlan). Peritoneal macrophages were obtained by adhering PEC to a polystyrene flask for 3–4 h after which remaining adherent cells were incubated with ice cold PBS (without calcium or magnesium) at 4 °C for 10 min. Cells were harvested with a cell scraper, then washed in RPMI and resuspended at 2 × 10^6^ ml^−1^.

### 2.2 Murine lymph node preparation

DO11.10 mice, with CD4^+^ T cells specific for the OVA_323–339_ peptide in the context of the MHC Class II molecule I-A^d^ [19] were obtained originally from N. Lycke, University of Göteborg, Sweden. This T cell receptor is recognized by the KJ1.26 clonotypic antibody. Single cell suspensions of lymph nodes from DO11.10 mice were obtained by passing tissues through 100 μm Nitex membrane (Cadish, London, UK) using a 5 ml syringe plunger. To enrich for lymphocytes in lymph node cell suspensions, cells were resuspended at 4 × 10^6^ ml^−1^ in complete RPMI, and cultured in 150 cm^3^ polystyrene flasks (Iwaki, Japan), for 2 h after which non-adherent cells were removed. Cells were washed three times in RPMI and resuspended at the desired cell concentration in complete RPMI with 25 mM HEPES.

### 2.3 Assessment of cell proliferation and cytokine production

Cells were cultured under conditions indicated in figure legends. Where indicated, transwells (0.2 μm membrane, Fisher Scientific, UK) separated cell populations. Cell proliferation was assessed by incorporation of [^3^H]thymidine (0.5 μCi/well) for the last 18 h of culture. Cells were harvested using a Betaplate 96-well harvester (Wallac Oy, Turku, Finland) and [^3^H]thymidine incorporation measured on a Betaplate liquid scintillation counter (Wallac). Concentration of cytokines in supernatants were estimated by ELISA. Paired antibodies (Pharmingen, Oxford, UK) were used in accordance with the manufacturer's recommendations. All assays were optimised and validated prior to use. Maxisorp 96-well plates (Nunc) were coated with capture antibody in 0.1 M NaHCO_3_ overnight at 4 °C. Plates were blocked for 2 h; samples and standards added for 2 h, followed by biotinylated secondary antibody for 1 h, then ExtrAvidin peroxidase (Sigma) conjugate for 45 min. Plates were developed with TMB (3,3′,5,5′-tetra-methylbenzidine, KPL, Gaithersburg, USA), and then read at 630 nm with a Dynex MRX II plate reader and analysed using Dynex Revelation 3.2 software (Dynex, Middlesex, UK). The levels of cytokine in supernatants were calculated by comparison with recombinant cytokine standards (R&D Systems, Abingdon, UK).

### 2.4 Hybridoma cells

The DO11.10-GFP hybridoma (kind gift of David Underhill, Department of Immunology, University of Washington, Seattle, Washington [20]) cells were grown in medium containing a 0.5 mg/ml geneticin. Co-cultures were established in which neutrophils or macrophages were incubated with 10 μg/ml OVA_323–339_ peptide and DO11.10-GFP hybridoma cells, in the ratio indicated in the figure legends. Cells were cultured for 14 h, washed, and then analysed by flow cytometry.

### 2.5 Flow cytometry

Aliquots of 1 × 10^6^ cells in 12 mm × 75 mm polystyrene tubes (Falcon BD, Oxford, UK) were resuspended in 100 μl FACS buffer (PBS, 2% FCS and 0.05% NaN_3_) containing Fc Block (Becton Dickenson, Oxford, UK) as well as appropriate antibody or isotype control. Neutrophils were identified with anti GR-1 (RB6-8C5, Caltag), macrophages with anti F4/80 (C1:A3-1, Caltag), and MHC Class II assessed (2G9, BD Biosciences), and T cells identified using anti CD4^+^ (RM4-5, BD) and a clonotypic antibody specific for the OVA_323–339_ TCR (KJ1.26). After washing, samples were analyzed using a FACSCalibur flow cytometer equipped with a 488 nm argon laser and a 635 nm red diode laser and analyzed using CellQuest software (both BD).

### 2.6 Statistical analysis

Statistical analysis was performed on data as indicated in the figure legends, using Minitab software.

## 3 Results

### 3.1 Purification of murine neutrophils

**Fig. 1:**
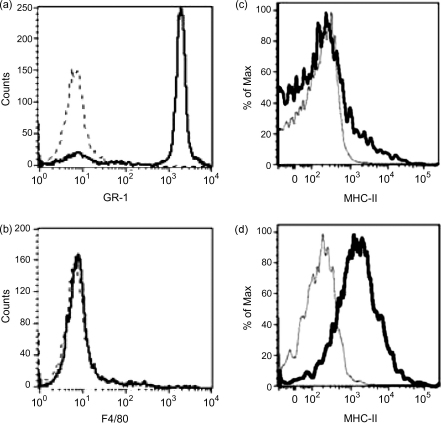
MHC Class II expression by peritoneal exudate cells. Purified murine neutrophils were greater than 85% GR1 positive (a), and less than 4% F4/80 positive (b). GR-1 positive neutrophils showed low levels of MHC Class II expression (c); F4/80 positive macrophages showed relatively higher MHC Class II expression (d). Data shown are representative of three independent experiments.

Neutrophils were purified from thioglycollate-induced peritoneal exudate cells (PEC), as there is a relative paucity of neutrophils in murine peripheral blood (data not shown). PEC were harvested 4 h post-thioglycollate administration, previously shown to be the peak of neutrophil recruitment, and after which an increasing proportion of macrophages are recruited [18]. Approximately 65% of thioglycollate-induced PEC were identified as neutrophils on the basis of GR1 staining (data not shown). PEC were further enhanced for neutrophils with a Ficoll gradient purification as previously described [18]. Following purification, samples were routinely 85% GR1 positive (Fig. 1a). Importantly, samples contained few macrophages (<4%) as determined by F4/80 staining (Fig. 1b). Cytospins of neutrophil preparations were prepared and demonstrated characteristic multilobed nuclei in >85% or cells (data not shown). Macrophage samples were 50–60% F4/80 positive cells (data not shown). Murine neutrophils, expressing GR-1, were found to express MHC Class II (Fig. 1c), albeit at low levels relative to F4/80 positive macrophages (Fig. 1d).

### 3.2 Murine neutrophils are able to present peptide antigen

**Fig. 2:**
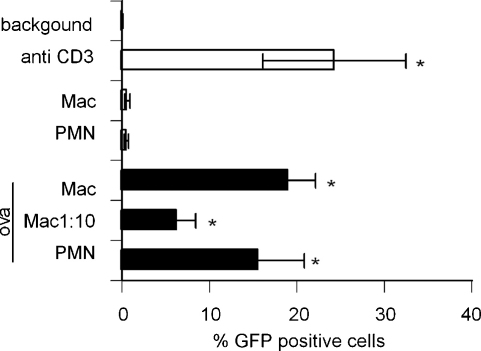
GFP expression by the DO11.10-GFP hybridoma following co-culture with APCs. Purified murine neutrophils (PMN) or macrophages (Mac) were cultured with 10 μg/ml OVA_323–339_ peptide and DO11.10-GFP cells for 14 h. Cells were washed and GFP expression was assessed by flow cytometry. ‘Background’ is a measure of GFP expression of DO11.10-GFP cells cultured with OVA_323–339_ alone; anti-CD3 was used as a positive control. Data are mean ± S.E.M. of three experiments. In each experiment, neutrophils were purified from thioglycollate-induced PEC pooled from 5 BALB/c mice. Macrophages were obtained from two similarly treated BALB/c mice. *p < 0.05 vs. background by Mann–Whitney *U* test.

The murine T cell hybridoma, DO11.10-GFP, is activated by OVA_323–339_ peptide in the context of I-A^d^, without any requirement for co-stimulation. The hybridoma does not respond to peptide alone (Fig. 2, ‘background’). Neutrophils or macrophages alone had no effect on GFP expression by DO11.10-GFP. However, neutrophils or macrophages loaded with OVA_323–339_ stimulated significant GFP expression by hybridoma cells when cultured at a 1:1 ratio. A dilution of macrophages was performed to estimate the contribution of contaminating macrophages to the results observed with neutrophil populations, demonstrating that at a ratio of 1 macrophage to 10 DO11.10-GFP cells, there was significantly less expression of GFP protein compared to that induced by neutrophils at a 1:1 ratio (Fig. 2). At a ratio of 1:100, there was no consistent GFP expression detected (data not shown). As there were <5% F4/80 positive macrophages contaminating the neutrophil samples, these titration experiments indicated that only a small proportion of the T cell hybridoma activation observed in the ‘neutrophil presentation’ assays could be attributable to macrophage contamination.

### 3.3 Neutrophils can drive naïve T cell proliferation and cytokine production

**Fig. 3:**
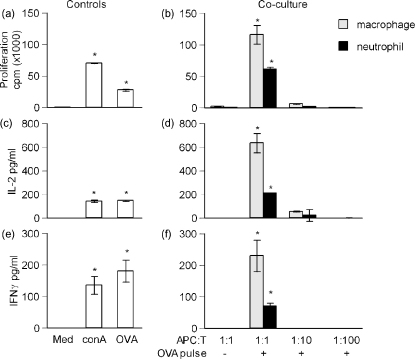
In vitro proliferation of DO11.10 lymph node cells. Single cell suspensions of lymph nodes were obtained from a DO11.10 transgenic mouse; neutrophils or macrophages were obtained as in Fig. 2. Cells were cultured for 72 h and proliferation was assessed by [^3^H] thymidine incorporation over the final 18 h of culture; cytokine production was assessed by ELISA of cell culture supernatants. DO11.10 lymph node cells were either cultured only with the stimuli indicated (a, c and e), or co-cultured with neutrophils or macrophages (b, d, and f). The ratio of APC to T cell in the culture, and whether the APC was pulsed with OVA_323–339_ prior to addition to the co-culture, is indicated on the *x*-axis. Data are mean ± S.E.M. of triplicate cultures. *p < 0.05 vs. medium by Student's *t*-test, representative of three similar experiments.

To investigate neutrophils antigen presentation in a co-stimulation dependent system, and the ability of neutrophils not only to present but also to prime T cells, neutrophils were co-cultured with primary T cells. Lymph nodes were obtained from DO11.10 transgenic BALB/c mice. Single cell suspensions of lymph nodes were allowed to adhere to plastic for 3 h to remove the majority of adherent antigen presenting cells, after which the population was routinely 70% CD3 positive (data not shown). The presence of remaining endogenous APCs in the lymph node facilitated a strong response to OVA_323–339_ peptide alone (Fig. 3a, c and e). Therefore, neutrophils and macrophages were pulsed with OVA_323–339_ for 2 h then washed three times to remove unbound peptide prior to the addition of transgenic T cells. Cells were co-cultured for 72 h at the ratios indicated in the figure legends. Neutrophils pulsed with OVA_323–339_-induced proliferation (Fig. 3b), IL-2 (Fig. 3d) and IFNγ (Fig. 3f) production. Low levels of IL-5 were detected in some experiments (data not shown). The neutrophil-induced responses were less than those evoked by macrophages at the same cell ratio. A titration of macrophages was performed to give an estimate of the contribution of contaminating cells, demonstrating that only a small proportion of neutrophil-induced T cell responses could be attributable to contaminating macrophages.

### 3.4 Cell contact dependence of neutrophil T cell interactions

**Fig. 4:**
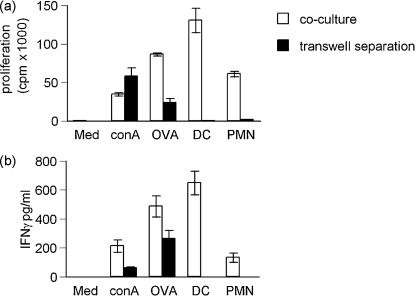
Antigen-specific proliferation and cytokine production was cell contact dependent. Single cell suspensions derived from lymph nodes of DO11.10 mice, were stimulated with Con A, OVA_323–339_, or APCs (DCs or neutrophils), which had been pulsed with OVA_323–339_. In parallel cultures, the stimulus was separated from the responding T cells by a transwell membrane. The stimuli were placed in the upper compartment, and (a) proliferation and (b) IFNγ production by the OVA_323–339_-specific T cells in the lower chamber was assessed. T cells did not respond when cultured with APCs alone (data not shown). Similarly, culturing T cells in the upper chambers resulted in no measurable response in the lower chambers. Data are mean ± S.E.M. of triplicate cultures and representative of two similar experiments.

To determine the relative contribution of cell contact dependent mechanisms and soluble factors in neutrophil-T cell interactions, cells were separated by 0.2 μm membranes placed between the cell populations, allowing diffusion of soluble proteins but preventing cell contact. The responding OVA_323–339_-specific T cells were not a 100% pure T cell population and contained some APCs, demonstrated by the lymph node response to OVA_323–339_ alone (Fig. 4a and b ‘OVA’). Thus, the transwells permitted investigation of whether lymph node-derived APCs were presenting soluble proteins derived from dead or degraded neutrophils. Bone marrow-derived dendritic cells (DC) were used as a positive control. Free OVA_323–339_ or ConA crossed the transwell membrane and stimulated cell proliferation and cytokine production (Fig. 4a). However, when antigen pulsed neutrophils were separated from the lymph node cells by the transwell, antigen-specific proliferation and cytokine production was reduced (Fig. 4a and b). Importantly, antigen pulsed cell cultures did not release sufficient free antigen to stimulate a response from the OVA_323–339_-specific T cells below (Fig. 4a). Thus, the antigen-specific T cell proliferation and cytokine production induced by neutrophil antigen presentation is cell contact dependent.

## 4 Discussion

Data presented here demonstrate that murine neutrophils are indeed capable of directly presenting antigen on Class II MHC and activating T cells independently of other APCs. The DO11.10-GFP hybridoma cells respond exclusively in a Class II restricted manner, and our data demonstrate that neutrophils, rather than contaminating cells, were mediating this response.

At comparable cell ratios, neutrophil-mediated T cell activation was lower than that mediated by macrophages, commensurate with reduced expression of MHC Class II by neutrophils. Although neutrophils have been reported to express co-stimulatory molecules, we detected only low levels of CD40 and were unable to consistently detect CD80 or CD86 in our neutrophil preparations (in contrast to the F4/80 positive macrophages which expressed CD40, CD80 and CD86; data not shown). Thus it is possible that: co-stimulatory molecule expression below the limits of detection of methods used here is sufficient for inducing a response, that T cells are responding in a co-stimulation independent manner, or that co-stimulation has occurred via alternative molecules.

Neutrophil antigen processing capacity was investigated using whole ovalbumin protein compared to OVA_323–339_. These studies indicated that these neutrophils did not process the ovalbumin effectively, demonstrated by a lack of response by DO11.10-GFP hybridoma (data not shown). An MHC Class I processing pathway within neutrophils was elegantly defined with *E. coli* transfected to express a MHC Class I restricted epitope of OVA. This demonstrated that neutrophils are capable of non-classical antigen processing for presentation on MHC Class I [17]. Although neutrophil enzyme mediated degradation of collagen generates a Class II epitopes (14), there is no current evidence of any Class II processing pathway. It has been proposed that as macrophage phagosomes are adequate for Class II antigen processing [21], therefore a parallel system may exist in neutrophils. A definitive investigation of murine neutrophil MHC Class II antigen processing awaits transfection of bacteria with OVA_323–339_, thus elucidating the effects on antigen processing of concomitant neutrophil activation by bacteria.

There is increasing evidence documenting the potential for neutrophils to acquire macrophage [22] or dendritic cell (3) characteristics, often in response to in vitro conditions. We document in vitro antigen presentation by in vivo activated neutrophils. It will be of interest to delineate whether such neutrophil behaviour can be observed strictly in vivo. Physiologically, the location of such neutrophil-T cell interactions is likely to be restricted to sites of chronic or established inflammation, for example persistent bacterial infection [15]. Neutrophils have been shown to degrade proteins to release antigens recognized by auto reactive T cells [16], and such antigens may then be transported to the draining LN by migrating DCs. Due to the neutrophils’ unique proteolytic activities, this may represent a mechanism of presentation of novel or cryptic antigens. Furthermore, there is evidence that neutrophils may passively acquire membrane proteins, including MHC, from other cells [23]. In our GFP-hybridoma studies (Fig. 2), this seems unlikely as the neutrophils were harvested from the peritoneum before there was significant in vivo macrophage migration, and immediately purified in vitro. However, this phenomenon may have contributed to the neutrophil mediated T cell activation in co-culture (Fig. 3).

There are overall fewer studies of murine neutrophils compared to human neutrophils, which is likely to be a reflection of the ease of acquiring human neutrophils that constitute 70% of circulating leucocytes, compared to limited quantity of murine peripheral blood available and the relative paucity of neutrophils, contributing only 20% of circulating leukocytes in rodents. These studies were only possible through use of rodent cells, as the TCR transgenic T cells absolutely control antigen specificity of response, which was central to this investigation of neutrophil-T cell interactions. However, the significantly different proportion of neutrophils in murine and human peripheral blood raises questions as to whether murine and human neutrophils perform parallel functions as is assumed. Our data document a novel, Class II restricted antigen presenting function of murine neutrophils, confirming findings in other human systems, and expanding our understanding of the role of the neutrophil in the adaptive immune response.
